# Impacts of Alternative Splicing Events on the Differentiation of Adipocytes

**DOI:** 10.3390/ijms160922169

**Published:** 2015-09-14

**Authors:** Jung-Chun Lin

**Affiliations:** School of Medical Laboratory Science and Biotechnology, College of Medical Science and Technology, Taipei Medical University, 250 Wu-Hsing Street, Taipei 11031, Taiwan; E-Mail: lin2511@tmu.edu.tw; Tel.: +886-2-2736-1661 (ext. 3330); Fax: +886-2-2732-4510

**Keywords:** adipogenesis, alternative splicing, splicing factor, transcriptome analysis

## Abstract

Alternative splicing was found to be a common phenomenon after the advent of whole transcriptome analyses or next generation sequencing. Over 90% of human genes were demonstrated to undergo at least one alternative splicing event. Alternative splicing is an effective mechanism to spatiotemporally expand protein diversity, which influences the cell fate and tissue development. The first focus of this review is to highlight recent studies, which demonstrated effects of alternative splicing on the differentiation of adipocytes. Moreover, use of evolving high-throughput approaches, such as transcriptome analyses (RNA sequencing), to profile adipogenic transcriptomes, is also addressed.

## 1. Introduction

Alternative splicing (AS) constitutes a prevalent mechanism in expanding the genetic diversity of eukaryotic cells [1]. Spatiotemporal expression profiles of AS transcripts substantially contribute to cell differentiation, specification, and organogenesis [2]. Approximately 90% of human genes generate more than one transcript by undergoing this meticulously controlled process [3,4], but further investigation is required to decipher the detailed mechanisms involved in alternative splicing. Nevertheless, tissue- and stage-specific splicing events are precisely manipulated by the interplay between splicing factors and corresponding *cis*-elements within transcripts [5].

Adipose tissues are endocrine organs that play an important role in energy homeostasis [6]. Adipocytes not only store lipids but also regulate energy expenditure by releasing a series of adipokines [7]. Two major types of adipocytes, white (WAs) and brown adipocytes (BAs), were first identified in mammals according to their macroscopic appearance [8]. Increase in the mass of white adipose tissues (WATs) that store triglycerides (TGs) leads to obesity. In contrast, brown adipose tissues (BATs) dissipate fatty acids in the form of heat to maintain the body temperature [9], which implies their therapeutic potential for combating obesity. Imbalanced energy homeostasis has led to a substantial rise in the worldwide incidence of obesity, a common origin of many metabolic diseases. Reprogramming splicing profiles constitutes an important mechanism which modulates tissue development, including adipogenesis. Alternatively spliced transcripts encode variants that exert different or even opposite effects on adipogenesis [10,11,12,13]. However, examples of splicing factors that regulate alterative splicing in differentiating adipocytes have rarely been documented. In this review article, we discuss recent studies regarding how splicing factors influence adipocyte differentiation by modulating alternative splicing events. Moreover, we focus on elucidating the molecular mechanisms of adipogenesis using advanced high-throughput technologies.

## 2. Overview of Alternative Splicing

**Figure 1:**
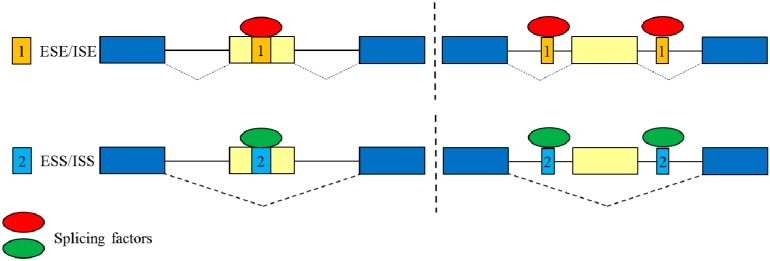
Regulation of alternative splicing events by the interplay between *trans*-splicing factors and *cis-*regulatory elements within pre-mRNAs. Square 1 represents ESE (exonic splicing enhancer) or ISE (intronic splicing enhancer); square 2 represents ESS (exonic splicing silencer) or ISS (intronic splicing silencer); dotted line is applied to separate the exonic or intronic regulatory elements; blue rectangles represent the conserved exons; yellow rectangles represent the regulated exons; red and green ovals represent different splicing factors.

Pre-messenger (m)RNA splicing is an essential process required for gene expression in eukaryotic cells. Correct recognition and base pairing of 5′ and 3′ splice sites is the critical step in the assembly of spliceosome. This enzymatic complex is mainly composed of five small nuclear (sn)RNAs and more than 150 associated proteins [14]. However, the activity of spliceosome on identifying 5′ or 3′ splice sites is widely affected by the interplay between numerous *trans*-acting factors and the corresponding *cis*-elements within the exonic and intronic regions [15]. The *cis*-elements are further classified into exonic and intronic splicing enhancers (ESEs and ISEs) or silencers (ESSs and ISSs) according to their influence on alternative splicing events [16]. The *trans*-factors which directly interact with the *cis*-elements promote or repress the assembly of spliceosomes, in turn manipulating the utilization of 5′ or 3′ splice sites (Figure 1). Ser/Arg rich (SR) proteins and heterogeneous nuclear ribonucleoproteins (hnRNPs) are two major groups of splicing factors. Many studies demonstrate that SR proteins mainly facilitate the utilization of alternatively spliced exons, whereas hnRNP proteins exert antagonistic effects and consequently interfere with the utilization of regulated exons. It is widely believed that the splicing profiles are reprogrammed with the relative expression of numerous splicing factors in nuclei, respectively [17].

## 3. Overview of Adipocyte Differentiation

Mesenchymal stem cells (MSCs) give rise to an adipocytic lineage through two major steps: an early commitment step and a terminal maturation step [18,19]. After the commitment phase, MSCs are unipotent and can only differentiate into an adipocytic lineage. During the terminal maturation step, adipocytic progenitors give rise to well-differentiated adipocytes responsible for lipid synthesis, transportation, expenditure, and production of adipokines which modulate energy homeostasis. A remarkable change in the gene expression profile was revealed to constitute the adipocyte-specific signaling cascade, which determines the phenotypic and functional signatures of mature adipocytes during the differentiation process. In summary, CCAAT/enhancer-binding family of proteins (C/EBPs), bone morphogenetic proteins (BMPs), peroxisome proliferator-activated receptors (PPARs), and interacting proteins play critical roles in the commitment and maturation steps of adipogenesis [20,21,22]. However, each type of adipocyte (WAs and BAs) arises from distinct MSC lineages and exhibits unique expression profiles of development-related transcription factors [23,24,25]. Therefore, identifying and understanding the molecular mechanisms that participate in the commitment and maturation steps of adipocytes will bring new insights toward developing therapeutic strategies to combat obesity, metabolism syndrome, insulin diabetes, and related diseases.

### 3.1. White Adipocytes

The best-known function of WATs is to store nutrients in the form of TGs for energy demands, such as starvation. WATs are primarily composed of WAs which contain unilocular fat droplets and few mitochondria. Transcriptional signaling that participates in WA differentiation is well studied. C/EBP family members, PPARγ, Krüppel-like factors (KLFs), and CREB are key factors of the WA-specific transcriptional cascade. Upregulated C/EBPβ and C/EBPδ proteins enhance expressions of PPARγ and C/EBPα in the early stage of the maturation phase [26,27,28,29]. PPARγ next constitutes a feedforward circuit with C/EBPα and C/EBPβ to further reinforce the terminal differentiation of WAs [30].

### 3.2. Brown Adipocytes

BATs mainly play a role in heat production in rodents and infants through non-shivering thermogenesis [31]. Apart from lipid storage, BAs are comprised of multilocular lipid droplets and abundant mitochondria. Canonical BAs share a developmental origin with skeletal muscle but presumably not with WAs [32]. Classical BAs arise from Myf5^+^ progenitors during prenatal development and subsequently populate interscapular BATs [33]. BMP family members substantially contribute to the development of distinct adipocytes. BMP2 and BMP4 are critical for the commitment and maturation steps of WATs [34,35]. BMP7 was demonstrated to trigger the development of BAs from both committed and uncommitted precursors [21,36]. BMP7 activates the expression of the master regulator of BAT development, PRDM16, which represses the development of WATs and skeletal muscle [37,38]. BMP7 promotes activation of the p38 mitogen-activated protein kinase (MAPK) pathway, which is essential for mitochondrial biogenesis in BATs [38].

## 4. Impacts of Alternative Splicing Events on Adipogenesis

### 4.1. Adipogenesis-Related Splicing Factors

#### 4.1.1. Src-Associated Substrate During Mitosis of 68 kDa (Sam68)

**Figure 2:**
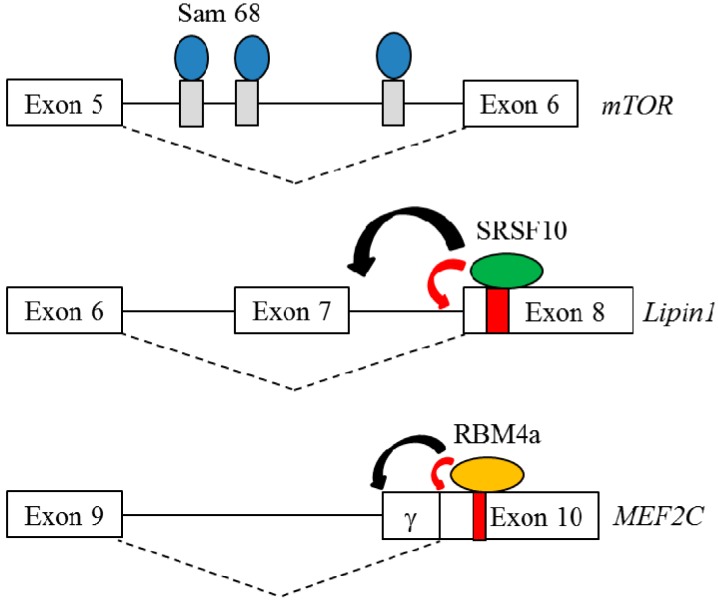
Schematic diagram represents the underlying mechanism for Sam68, SRSF10 and RBM4a-regulated AS events during adipogenesis. Blue ovals represent Sam68 proteins. Green ovals represent SRSF10 proteins and orange ovals represent RBM4 proteins; Gray and red rectangles represent the binding elements of Sam68, SRSF10 and RBM4; Red arrowhead represents the activation of splice site, whereas black arrowhead represents the repression of splice site.

Sam68 belongs to the larger class of the heteronuclear ribonucleoprotein particle K (hnRNP K) homology (KH) domain family of RNA-binding proteins [39]. Sam68 was demonstrated to regulate alternative splicing by recognizing the U(U/A)AA sequence neighboring alternatively spliced exons [40]. *Sam68^−/−^* mice exhibited increased energy consumption and resistance to dietary-induced obesity, implying its potential role in lipid expenditure [41]. A profile of genome-wide exon utilization was analyzed using RNA extracted from *Sam68^−/−^* and wild-type (WT) WATs. A mammalian target of rapamycin (mTOR) was characterized as one candidate of Sam68-regulated splicing events in WAs [42]. Sam68 ablation resulted in a relatively high level of labile *mTOR*^+intron 5^ transcripts, which contained a frameshift-induced premature termination codon (PTC) [42]. The PTC-harboring transcripts are detected via pioneer round translation and subsequently degraded through Nonsense-mediated mRNA decay (NMD) which functions as a quality control mechanism to eliminate truncated and deleterious isoform to cells [43]. Figure 2 shows the underlying mechanism for the Sam68-enhanced splicing of *mTOR* intron 5 [42]. Downregulation of mTOR subsequently led to reduced phosphorylation of ribosomal protein S6 and Akt, which abrogated the effect of mTOR-mediated signaling on WAT development. Recently, Sam68 was demonstrated to modulate the splicing profile of ribosomal S6 kinase (S6K), which was involved in the mTOR signaling [44]. Ablation of Sam68 resulted in the generation of S6Kb1-002 transcript and the encoded S6K1-p31 protein, which is absent in the wild-type adipocytes. Sam68 and serine/arginine-rich splicing factor 1 (SRSF1) constituted the regulatory mechanism that controlled the expression of S6K1-p31 variant by competing the binding to *S6K1* intron 6. RNAi-mediated silencing of S6K1-p31 protein partially restored the differentiation of *Sam68^−/−^* preadipocytes. Consistently, the presence of overexpressing S6K1-p31 variant mediated the differentiation defect in NIH3T3-L1 cells, which suggested the suppressive effect of S6K1-p31 variant on adipogenesis [44]. These findings indicate that Sam68 is required to prevent the expression of S6K1-p31 in adipocytes for adipogenesis to occur. The influence of Sam68 on mTOR signaling and the altered splicing profile in *Sam68^−/−^* WATs suggested that Sam68 may be a key regulator of WAT-associated splicing events, which were essential for the WAT development.

#### 4.1.2. Serine/Arginine-Rich Splicing Factor 10 (SRSF10)

SRSF10 was demonstrated to be a sequence-specific splicing factor [45]. GA-rich hexamers (e.g., GAAAGT/GAAGAA and AGAAA) were identified as the potential binding sites of SRSF10 within the alternatively spliced or flanking exons by using the SELEX approach [46]. Embryonic lethal was the major phenotype of *SRSF10^−/−^* due to cardiac defects, which suggested the potential influence of SRSF10 on cardiac tissue-specific splicing events [47]. In addition to the heart, the *SRSF10^−/−^* mice exhibited impaired development of axillary subcutaneous WATs compared to WT mice [48]. The result of a transcriptome analysis using RNA extracted from *SRSF10^−/−^* and WT MEF cells identified and further validated 16 SRSF10-regulated splicing events. Several SRSF10-regulated candidates, including *ACLY* [49], *Axin1* [50], *UPF1*, and *Lipin1* [51] were implicated in adipogenesis. Among the candidates, alternatively spliced transcripts of *Lipin1* encoded two isoforms, which exerted distinct functions in adipogenesis of WAs [52]. Direct binding of SRSF10 to *Lipin1* exon 8 induced a relatively high level of *Lipin*^−exon 7^ transcripts (*Lipin1α*) which was required for the early differentiation of preadipocytes (Figure 2; [48]), whereas the presence of *Lipin1*^+exon 7^ transcripts (*Lipin1β*) contributes to the lipid accumulation of mature WATs [48]. Overexpression of Lipinα partially rescued the impaired development of WAs in *SRSF10^−/−^* mice. Relatively high expression of *PKM2*, the alternatively spliced transcript of *PKM* gene were also noted in *SRSF10^−/−^* adipocytes, which suggested a potential role of SRSF10 in differentiation and metabolism of white adipogenesis.

#### 4.1.3. Fat Mass and Obesity-Associated Protein (FTO) and SRSF2

FTO protein was identified as an obesity gene which belongs to the non-heme Fe(II)/dioxygenases (AlkB family). FTO-knockin or knockout mice both exhibited impaired metabolism homeostasis and body mass, suggesting its pivotal role in adipogenesis [53]. *In vivo* studies indicated that FTO specifically mediated the demethylation of RNA N6-methyladenosine (m6A), which is essential for the posttranscriptional regulation, including RNA splicing [54,55]. Upon FTO depletion, the adenosine resides within the exonic regulatory elements (e.g., ESEs or ESSs) flanking the splice site was hypermethylated compared to the adjacent intronic sequences [54]. *In vitro* binding assays showed that the interactions between SRSF2 and ESEs were manipulated with the methylation of ESE-harboring adenosine, which may function as a novel signal to splicing factors. FTO ablation was demonstrated to reprogram the splicing profiles of *Runt-related transcription factor 1* (*RUNX1T1*) gene, which generated the exon 6-included and exon 6-skipped transcripts [54]. Gradual reduction of FTO protein with a concomitant decrease in the *RUNX1T1*^−exon 6^ transcripts were observed during the adipogenesis, which was consistent with the relative low expression of *RUNX1T1*^−exon 6^ transcripts in FTO-depleted preadipocytes [54]. In contrast, the adipogenesis was exhibited in the presence of overexpressing RUNX1T1^+exon 6^ isoform, of which the expression was upregulated in the differentiating adipocytes. Nevertheless, FTO-mediated adenosine methylation functions as a new signal to the regulation of alternative splicing.

#### 4.1.4. Zinc Finger Protein 638 (ZNF638)

Zinc finger proteins constitute complex families that mainly function as transcription factors [56]. ZNF638 is a multidomain protein harboring Arg/Ser-rich (RS) domain, RNA recognition domain and two zinc finger motifs [57]. Gradual increase in ZNF638 was observed in the differentiating adipocytes, which implied its potential effect on adipogenesis [58]. Overexpressing ZNF638 and C/EBP proteins constituted a feedforward pathway that enhanced the promoter activity of *PPARγ* gene [57]. Immunostaining assay showed the colocalization of ZNF638 and splicing factors in nuclear speckles [58]. The interplay between ZNF638 and splicing factors reprogrammed the splicing profiles of *Lipin1* and *Nuclear receptor co-repressor 1* (*NCoR*) genes. Presence of overexpressing ZNF638 programmed the expression profiles of Lipin1 and NCoR isoforms that participated in the different stages of adipogenesis. Nevertheless, the nuclear localization and function domain suggested the potential effect of ZNF638 protein on posttranscriptional controls during adipogenesis.

#### 4.1.5. RNA-Binding Motif Protein 4a (RBM4a)

RBM4a functions as a multifunctional RNA-binding protein that is involved in splicing regulation and translation control [59,60]. RBM4a reportedly reprograms the tissue-specific splicing network, which modulates development of muscles and pancreatic β-islets [61]. *RBM4a^−/−^* mice exhibited metabolic phenotypes, including hyperglycemia, hypoinsulinemia, hyperlipidemia, and a reduced mass of interscapular (i) BATs [62,63]. A gradual increase in RBM4a protein was observed during the development of BATs and in differentiating primary adipocytes and C3H10T1/2 cells [62,63], which mediated the reprogramming of BA-associated splicing events. Elevated RBM4a reprogrammed the splicing profiles of *IR*, *PPARγ*, and *Pref-1* genes, which participated in the different stages of adipogenesis [62]. Upregulation of RBM4a relieved the repressive effect of Pref-1 on adipogenesis by enhancing the relative level of *Pref-1C* and *Pref-1D* transcripts, encoding the soluble membrane isoforms [12,62], whereas the large *Pref-1A* and *Pref-1B* transcripts functions as the adipogenic repressors [12]. RBM4a enhanced the expression of *insulin receptor B* (*IR-B*) transcripts, which are dominantly expressed to mediate BA-associated signaling pathways in differentiating BAs [13,62]. RBM4a upregulation was highly relevant to the increase in *PPARγ2* transcripts and PRDM16 and BMP7 that all facilitated the BA-related transcriptional networks [62,63]. However, the underlying mechanism for RBM4a-induced expression of *PPARγ2* transcripts is further investigated. In our previous study, the direct binding of overexpressing RBM4a to *MEF2C* exon 10 was demonstrated to enhance the relative level of the MEF2Cγ-isoform (Figure 2; [63]) which induced the expression of BA-specific factors, including RBM4a [63]. It is intriguing that RBM4a was observed to autoregulate its own splicing profile, subsequently enhancing transcription of full-length *RBM4a* mRNA [62]. Recently, we demonstrated the regulatory activity of RBM4a on reprogramming the splicing profiles of *FGFR2* and *PKM* gene, which contributed to the development and function of BAs. These results substantially indicate the wide influence of RBM4a on the development and function of BAs. The adipogenesis-related splicing regulators are summarized in Table 1.

**Table 1:** Distinct splicing factors modulate a set of adipogenesis-related AS events.

Splicing Factor or Regulator	AS Event	Adipocyte	Biological Signatures	References
Sam68	*mTOR*	WAT	Promote adipogenesis	[41,42]
SRSF10	*Lipin1*	WAT	Differentiation/Lipid storage	[48,51,52]
SRp40	*PPARγ*	Pre-adipocyte	Promote adipogenesis	[64,65]
SRSF2 & FTO	*RUNX1T1*	WAT	Promote adipogenesis	[54,55]
RBM4a	*PPARg*, *Pref-1 INSR*, *MEF2C*	BAT	Enhance differentiation and metabolismof BAs	[12,13,62,63]

### 4.2. Adipocyte-Related Alternative Splicing Events

#### 4.2.1. Nuclear Receptor Co-Repressor 1 (NCoR)

Alternative splicing constitutes a molecular mechanism for manipulating the transcriptional plasticity of numerous transcriptional factors or interacting cofactors in activating or repressing target genes [63]. Nuclear receptor co-repressor 1 and 2 (also known as NCoR and SMRT, respectively) are well-studied corepressors that participate in transcriptional regulation of lipid and glucose metabolism [66]. *NCoR* and *SMRT* genes are documented to encode various isoforms, which differ in their receptor interaction domains (RIDs) through alternative splicing mechanisms [67]. NCoR and SMRT share overlapping functions of mediating the repressive effects of a broad diversity vertebrate transcription factors [68]. Hormone-related nuclear receptors, including thyroid hormone receptors (TRs), retinoic acid receptors, PPARs, liver X receptors (LXRs), and estrogen receptors, target regulated genes and recruit the NCoR or SMRT corepressors by binding to different RIDs [69,70,71,72]. The remaining regions of SMRT and NCoR in turn recruit histone deacetylase 3 (HDAC3), TBL/TBLR-1, or GPS2, that constitute the molecular mechanism required for transcriptional regulation [73]. The PPAR, TR, and LXR families contribute more to the transcriptional regulation of the development of metabolic tissues, such as adipocytes, than to the metabolism of lipid and glucose [74,75]. The *NCoR*^+exon 37b^ transcript encodes three RID-containing NCoRω variants that are mainly distributed in the brain, testes, and progenitor cells of WATs [76]. Reciprocally, exclusion of *NCoR* exon 37b results in generation of the NCoRδ isoform that is predominantly expressed in the heart, spleen, lungs, skeletal muscles, and WATs [76]. The NCoRω-to-NCoRδ switch was noted in differentiating adipocytes. *NCoRω^−/−^* mice exhibited the adiposity phenotype [77], whereas the *in vitro* functional assays clearly illustrated that overexpressing NCoRδ enhanced the development and function of white adipogenesis, such as lipid accumulation. Despite this, the mechanistic understanding underlying the utilization of *NCoR* exon 37b is still largely uncharacterized.

#### 4.2.2. Protein Kinase Cδ (PKCδ)

PKCδ is a serine-threonine kinase, which functions as an important modulator of cellular apoptosis [78]. Several apoptosis-related genes, including *MCL-1* and *PKCδ*, encoding alternatively spliced variants, which retain opposite effects [79,80]. The relative expression of PKC*δ* spliced variants, PKCδI and PKCδII, manipulate the cellular fate. PKCδI serves the apoptotic factor, whereas the presence of PKCδII enhances the cellular viability [81]. *PKCδII* transcripts are generated by utilizing an alternative downstream 5′ splice site of *PKCδ* exon 9, resulting in an insertion of 26 amino acids which interrupts the caspase-3 recognition site [81]. It is widely documented that the expressions of PPARγ and C/EBP program transcriptional profiles that promote the differentiation of primitive precursor cells toward maturity [30]. However, preadipocytes are more susceptible than mature adipocytes to apoptosis [82]. The gradual increase in the relative level of PKCδII, the antiapoptosis variant, was noted during the differentiation of NIH3T3-L1 cells [81]. The presence of PKCδII could constitute a mechanism for rendering mature adipocytes resistant to apoptosis. A synthetic compound was recently demonstrated to specifically abolish the relative expression of PKCδII, which, in turn, reduced the differentiation of NIH3T3-L1 cells [81,82,83,84]. It has been revealed that the overexpressing transformer 2β (Tra2 β) enhanced the utilization of authentic 5′ splice site of *PKCδ* exon 9, which consequently enhanced the relative level of *PKCδI* transcripts [85]. The diverse functions of PKCδ isoforms indicated the wide influence of alternative splicing events on the differentiation and termination of adipogenesis.

#### 4.2.3. Cholesteryl Ester Transfer Protein (CETP)

The CETP plays a pivotal role in cholesterol transport from the periphery to the liver for clearance [86]. Previous studies mainly focused on non-synonymous single-nucleotide polymorphisms (SNPs) that affect its physiological function [87]. However, the *CTEP* gene was documented to generate variants through alternative splicing mechanisms [88]. The exon 9-skipped CTEP was demonstrated to interact with its full-length variant, which acted in a dominant negative fashion when interfering with its secretion from the endoplasmic reticula (ER) [89]. A relatively high expression of the exon 9-skipped *CTEP* transcript was associated with two SNPs with minor allele frequencies, which reside within intron 8 (rs9930761) and exon 9 (rs5883). Even though the presence of rs5883 SNP alone could drive skipping of *CTEP* exon 9, the presence of these two SNPs had an additive effect on expressions of exon 9-skipped *CTEP* transcripts [89]. The stable overexpression of CTEP substantially diminished TG synthesis and inversely enhanced the turnover rate of TG without altering the expression of adipocyte-related factors [90,91]. Eventually, the overexpressing CTEP resulted in the formation of small and metabolically active lipid droplets in liposarcoma cells [90]. Interest is building in examining the SNP-coupled alternative splicing of the *CTEP* involved in the function of BAs in terms of fat metabolism and energy homeostasis, although more investigation is required to prove the inference.

#### 4.2.4. Peroxisome Proliferator-Activated Receptor γ (PPARγ)

**Figure 3:**
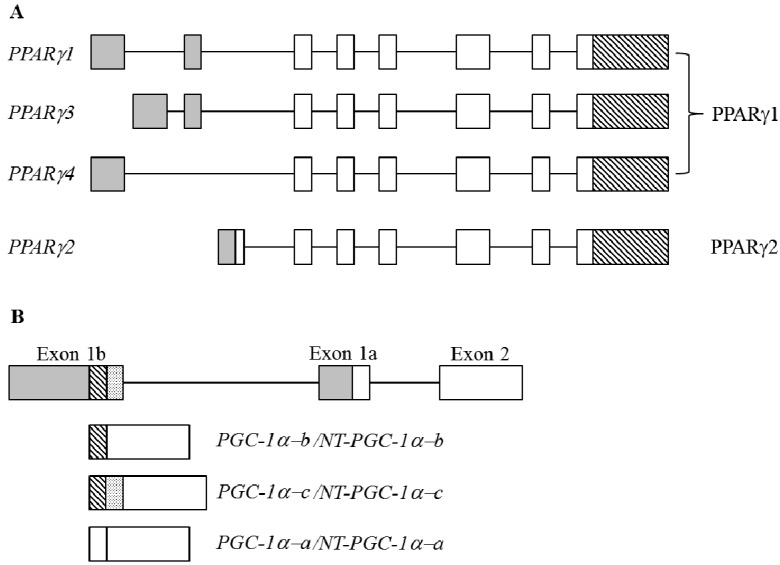
(**A**) Schematic diagram represents the *human* *PPARγ* gene and alternatively spliced transcripts. The gray rectangles with different sizes represent the 5′ untranslated regions (UTRs), and the backslash rectangles represent the 3′ untranslated regions (UTRs). The open rectangles represent the coding exons; (**B**) Schematic diagram shows the composition of N-termini of *murine PGC-1α* gene. The gray, backslash and dotted rectangles represent the different 5′ untranslated regions (UTRs), and the open rectangles represent the coding exons.

PPARγ is the best-characterized member of PPARs, belonging to the hormone ligand-dependent nuclear receptor superfamily [92]. PPARγ regulates the expression of numerous genes that are widely involved in glucose, lipid and cholesterol metabolism, cell proliferation, and tissue development [93,94]. PPARγ plays a pivotal role in regulating transcriptional events that participate in the onset stage of adipogenesis [30]. Defects in PPARγ were reported to result in different pathological conditions in metabolic syndrome, including insulin resistance, obesity, dyslipidemia, and hypertension, and largely increase the risk of type 2 diabetes, cardiovascular diseases, and cancer [95,96]. The human PPARγ gene consists of nine exons and transcribes four major transcripts (*PPARγ1*, *PPARγ2*, *PPARγ3*, and *PPARγ4*) through the use of differential promoters and alternative splicing mechanisms [97]. Despite these transcripts being composed of different 5′ untranslated regions (UTRs) and six coding exons, the *PPARγ* transcripts encodes two variants. *PPARγ1*, *PPARγ3*, and *PPARγ4* encode the PPARγ1 isoform that is ubiquitously expressed in the liver, heart, skeletal muscles, and WATs, whereas the *PPARγ2* transcript encodes the PPARγ2 isoform which contains 28 additional amino acids at the N-terminus and is enriched in adipose tissues (Figure 3A; [98]). *In vitro* functional assays indicated that both PPARγ1 and -2 are essential for adipogenesis, and more-relevant adipogenic activity for PPARγ2 was revealed in brown adipogenesis [99,100].

The physiological role of PPARγ in adipocyte differentiation was evaluated in detail, but the molecular mechanism regarding the alternative splicing of *PPARγ* remains largely unknown. Relatively high level of *PPARγ2* was noted to be relevant to the upregulated SRp40 expression in nuclei of differentiating NIH3T3-L1 cells, which was modulated by the presence of long noncoding (lnc)RNA *NEAT1* [64]. Overexpressing *NEAT1* transcripts potentiated the Clk1-mediated phosphorylation of SRp40, which facilitated the expression of *PPARγ2* transcripts in preadipocytes [64,65]. However, the expression of *NEAT1* transcripts was varied during the adipogenesis and therefore more functional assays are required to decipher relationships between *PPARγ* gene, SRp40 and *NEAT1* RNAs.

#### 4.2.5. PPARγ Coactivator 1α (PGC-1α)

BA-enriched PGC-1α was identified as a transcriptional co-factor of PPARγ, which functions as a key factor in oxidative phosphorylation and regulates adaptive thermogenesis toward cold stress and starvation [101]. Therefore, the expression and physiological activity of PGC-1α are modulated by environmental and nutritional conditions, which program transcriptional networks regarding cell adaptation [102]. The intron 6-retention of *PGC-1α* pre-mRNA resulted in an alternatively spliced transcript which encodes a shorter isoform, containing the N-terminal activation and interacting domains but which was missing all other domains within 268~797 amino acids of the full PGC-1α N-terminal truncated PGC-1α (NT-PGC1α) (Figure 3B; [103]). The specific roles of *PGC-1α* transcripts remain largely unknown due to their synchronous increase and equivalent activity in coupling with related transcriptional regulation of adaptive thermogenesis. Two novel *PGC-1α* transcripts (BB853729: PGC-1α-b and AW012094: PGC-1α-c) were recently identified in murine expressed sequencing tag (EST) databases, the expressions of which were also induced by cold stimuli in BAs and skeletal myocytes [104]. These transcripts were generated by splicing a non-conventional exon 1b at different 5′ splice sites to the conserved exon 2. The alternatively spliced PGC-1α-b and PGC-1α-c contained different N-termini which were four or 13 amino acids shorter than that of PGC-1α derived from the conventional exon 1a [104]. These variants all retained transcriptional activity and cellular localization similar to those of the full-length PGC-1α. However, cold exposure mediated a more-substantial increase in exon 1b-derived transcripts than those of conventional exon 1a-containing *PGC-1α* in BAs [104]. A fundamental yet undecipherable question is whether the induction of exon 1b-derived transcripts are involved in the tissue-specific or stimuli-related regulation.

#### 4.2.6. Mitochondrial Oxodicarboxylate Carrier (ODC)

ODC was demonstrated to participate in the transport of 2-oxoadipate and 2-oxoglutarate in distinct eukaryotic cells [105]. Mice ODC genes generate two major transcripts (*ODC* and *ODC-AS*) through the use of differential 5′UTR and the first exon [106]. ODC protein is ubiquitously observed in distinct tissues, whereas the expression of *ODC-AS* transcript was enriched in adipose tissues and iris [106,107]. Depletion of ODC-AS was documented to impair the lipid accumulation of differentiating adipocytes. Intriguingly, cold exposure substantially reduced the expression of *ODC-AS*, but not *ODC* transcripts in BATs, which may further suggest the potential effect of ODC-AS protein on the homeostasis of lipid metabolism [106], although the molecular mechanism involved in the alternative splicing of *ODC* gene is still largely unknown as described in previous genes. The adipogenesis-related splicing events are listed in Table 2.

**Table 2:** Exon usage and biological relevance of adipocyte-related AS events.

Gene	AS Region	Adipocyte Isoform	Biological Signatures	References
*mTOR*	Intron 5	Excluded	Promote white adipogenesis	[42]
*Lipin 1*	Exon 7	Included/Excluded	Differentiation/Lipid storage	[48,51,52]
*INSR*	Exon 11	Included	Promote brown adipogenesis	[13,62]
*MEF2C*	Alternative 3′ splice site of Exon 10	Distal	Promote brown adipogenesis	[63]
*NcoR*	Exon 37b	Included/Excluded	Proliferation/Differentiation	[66,67,68,69,70,71,72,73,74,75,76,77]
*PKCδ*	Alternative 5′ splice site of Exon 9	Proximal	Promote adipogenesis	[30,78,79,80,81,82,83,84,85]
*CETP*	Exon 9	Included	Promote lipid metabolism	[86,87,88,89,90,91]
*RUNX1T1*	Exon 6	Included	Promote white adipogenesis	[54]
*ODC*	Exon 1, 2 and 3	Selection of exon 2 and 3	Promote lipid accumulation	[105,106,107]

## 5. High-Throughput Methods Applied to Tissue-Specific Alternative Splicing

It is known that spatiotemporal splicing events constitute a mechanistic control in the development of a wide range of tissues and cells [3,4]. The development of high-throughput technologies largely expanded understanding of alternative splicing events in the last decade. It is helpful to investigate the mechanistic regulation of alternative splicing events with the basic principles of these approaches. Here, we summarize some high-throughput methods that are widely conducted on a genome-wide scale. Microarrays were an early genome-scale approach applied to alternative splicing studies [108]. To identify alternative splicing events, oligonucleotides that probed exon-exon junctions were designed for splicing-sensitive microarrays. The signal intensity of the hybridized probes can reflect the relative level of utilized exons using sophisticated algorithms [109]. However, the application of microarray platforms to *de novo* identification is highly restricted.

Deep RNA sequencing technology is an advanced method for genome-wide alternative splicing analyses [3]. Complementary (c)DNA libraries constructed from poly(A) enriched RNA pools are amplified and sequenced from the paired ends to generate short sequence tags, also known as “reads”. Large numbers of these reads are then mapped to the reference genome and splice site-aligned reads can reveal the presence of spliced transcripts. Compared to microarrays, RNA sequencing analysis is able to identify novel transcripts by aligning the amplified reads to the updated reference sequence of different species in an unbiased manner [110]. Moreover, the gene expression and isoform profile can be globally analyzed in one RNA sequencing analysis without cross-hybridization issues. The new sequencing platform, such as HiSeq-4000 (Illumina) substantially expends the reads number and coverage rate, which led to the identification of novel transcripts with relatively low expression levels. The high-throughput reverse-transcription polymerase chain reaction (RT-PCR) was developed to efficiently evaluate the results of RNA sequencing [111].

Experimental results of RT-PCR have confident percent spliced-in (PSI, the ratio of transcripts including alternatively spliced exons) values, which should be highly relevant to RNA-Seq data [112]. However, design of RT-PCR analyses for AS events is a time-consuming process that should be manually conducted. Recently, a PrimerSeq software has been developed for systematically designing the PCR primer sets of AS events [113]. In brief, PrimerSeq program can incorporate both original RNA-seq data (FASTA files), or pre-defined gene and transcript annotations files (GTFs). PrimerSeq next systematically designs PCR primer pairs for individual AS events on suitable flanking exons that are pre-specified or automatically selected [113]. Although the RT-PCR analysis should be conducted manually, these methods can complement each other in globally identifying and evaluating alternative splicing profiles. Moreover, the high-throughput RT-PCR method has the benefit of avoiding experimental bias due to spliced transcripts with high expression levels, and alternatively spliced transcripts can be quantified in a broad range.

The interplay between splicing factors and *cis*-elements constitutes the molecular mechanism, which programs the splicing profile in a spatiotemporal manner [5]. To further investigate the mechanistic regulation in tissue- or stage-specific alternative splicing events, it is necessary to directly map an RNA-binding protein (RBP) and its regulated candidate. To identify specific targets of a splicing factor, the associated RBP complex is first immunoprecipitated from cell lysates, and bound transcripts are then purified and subjected to an RNA sequencing analysis. After aligning the amplified reads back to the corresponding reference sequence, the potential binding site of the RBP can then be inferred using algorithms. A cross-linking step is usually performed to prevent loss of low-affinity but specific RNP complexes following cell extraction. Several methods have been developed in this area. The binding between proteins and RNAs is crosslinked using UV irradiation, followed by the cross-linking immunoprecipitation (CLIP)-high-throughput sequencing (Seq) analysis [114]. In photoactivatable-ribonucleoside-enhanced (PAR)-CLIP, photoreactive ribonucleotide analogs are incorporated into transcripts before UV treatment [115]. The result of CLIP-Seq can be used for the construction of “RNA maps” by combining with alternative splicing profiles, which correlates the responsive elements with different spliced transcripts upon manipulating specific splicing factors [116].

## 6. Conclusions and Perspectives

Imbalanced energy homeostasis leads to accumulation of lipid in WAs that principally causes obesity. In contrast, the high metabolic activity of BAs suggests its potential effect for combating obesity and metabolic diseases. Cold environment or exercise induces browning of WAs to brown adipocyte-like cells (so-called beige/brite cells) as abundant clusters which have both morphological and biological features of classical brown adipocytes in white adipose tissues of adult animals. A thorough understanding of the mechanisms underlying adipogenesis is critical for obesity management. In this review, recent progress in analyzing alternative splicing events and their potential influence in the development of white or brown adipocytes were summarized. These findings constitute a network in which alternative splicing events are integrated in transcriptional and post-transcriptional regulation. However, the underlying mechanisms for the adipogenesis-related alternative splicing events are mostly uncharacterized. With the progression of high-throughput technologies, such as RNA sequencing, new insights were gained into the mechanistic regulation by which alternative splicing networks participate in adipogenesis on a genome-wide scale. Additionally, identification of splicing factors that regulate adipocyte-specific splicing events using high-throughput approaches will be helpful for defining comprehensive strategies for understanding adipogenesis and the development of therapies to combat obesity and metabolic diseases.
